# External validation of the Bayesian Estimated Tools for Survival (BETS) models in patients with surgically treated skeletal metastases

**DOI:** 10.1186/1471-2407-12-493

**Published:** 2012-10-25

**Authors:** Jonathan Agner Forsberg, Rikard Wedin, Henrik CF Bauer, Bjarne H Hansen, Minna Laitinen, Clement S Trovik, Johnny Ø Keller, Patrick J Boland, John H Healey

**Affiliations:** 1 Regenerative Medicine, Naval Medical Research Center, Silver Spring, MD, USA; 2 Orthoapedic Oncology, National Military Medical Center, Bethesda, MD, USA; 3 Department of Molecular Medicine and Surgery, Section of Orthopaedics and Sports Medicine, Karolinska University Hospital, Karolinska Institutet, Stockholm, Sweden; 4 Department of Orthopaedics, Aarhus University Hospital, Aarhus, Denmark; 5 Division of Orthopaedics and Traumatology, Tampere University Hospital, Tampere, Finland; 6 Department for Orthopaedic Rehabilitation, Haukeland University Hospital, Bergen, Norway; 7 Department of Surgery, Orthopaedic Service, Memorial Sloan-Kettering Cancer Center, 1275 York Avenue, New York, NY, 10065, USA

**Keywords:** Bayesian analysis, Skeletal metastasis, Prognostic model, Postoperative survival

## Abstract

**Background:**

We recently developed two Bayesian networks, referred to as the Bayesian-Estimated Tools for Survival (BETS) models, capable of estimating the likelihood of survival at 3 and 12 months following surgery for patients with operable skeletal metastases (BETS-3 and BETS-12, respectively). In this study, we attempted to externally validate the BETS-3 and BETS-12 models using an independent, international dataset.

**Methods:**

Data were collected from the Scandinavian Skeletal Metastasis Registry for patients with extremity skeletal metastases surgically treated at eight major Scandinavian referral centers between 1999 and 2009. These data were applied to the BETS-3 and BETS-12 models, which generated a probability of survival at 3 and 12 months for each patient. Model robustness was assessed using the area under the receiver-operating characteristic curve (AUC). An analysis of incorrect estimations was also performed.

**Results:**

Our dataset contained 815 records with adequate follow-up information to establish survival at 12 months. All records were missing data including the surgeon’s estimate of survival, which was previously shown to be a first-degree associate of survival in both models. The AUCs for the BETS-3 and BETS-12 models were 0.79 and 0.76, respectively. Incorrect estimations by both models were more commonly optimistic than pessimistic.

**Conclusions:**

The BETS-3 and BETS-12 models were successfully validated using an independent dataset containing missing data. These models are the first validated tools for accurately estimating postoperative survival in patients with operable skeletal metastases of the extremities and can provide the surgeon with valuable information to support clinical decisions in this patient population.

## Background

Accurate, personalized survival estimates are important for patients with metastatic disease, partly because they can help guide surgical decision-making [1,2]. Importantly, survival estimates can help identify not only which patients may benefit from surgery but also which surgical procedure may be most appropriate. Both features are critical in the effort to avoid under- or overtreatment of the disease. Prognostic variables are generally considered favorable or unfavorable and include information based on oncologic diagnosis [3,4], extent of disease [5], the patient’s performance status [6], and basic laboratory assessments [7].

**Figure 1:**
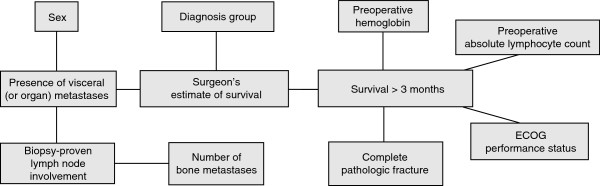
**BETS-3 model structure.** As shown, there are 5 first-degree associates of 3-month survival: surgeon’s estimate of survival, preoperative hemoglobin concentration, preoperative absolute lymphocyte count, ECOG performance status, and presence of a complete pathologic fracture.

**Figure 2:**
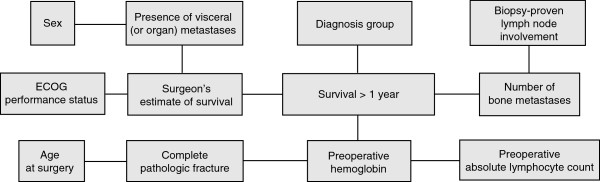
**BETS-12 model structure.** As shown, there are four first-degree associates of 12-month survival: surgeon’s estimate of survival, preoperative hemoglobin concentration, number of bone metastases, and primary oncologic diagnosis.

To better understand the relationships and relative importance of prognostic variables in patients with skeletal metastases, we previously analyzed readily available clinical data on a particular subset of these patients. Using a fully machine-learned algorithm, we developed two Bayesian classifiers to estimate the likelihood of survival at 3 and 12 months following the surgical treatment of skeletal metastases [4]. These clinical decision support models are referred to as the Bayesian Estimated Tools for Survival—the BETS-3 and BETS-12 models (Figures 1 and 2), respectively. The 3- and 12-month time points were chosen because they are widely considered useful for orthopaedic surgical decision making [8-10]. Specifically, when surgical stabilization is deemed necessary, shorter life expectancies are thought to warrant less-invasive stabilization procedures, such as intramedullary or plate fixation, that do not require prolonged rehabilitation periods. Accordingly, longer life expectancies may warrant more durable reconstruction procedures, using endoprostheses, which are associated with significant operative morbidity and longer rehabilitation times [8-10]. We developed two models because Bayesian classifiers are not well suited to provide discrete estimates in time, but rather probabilities of a particular outcome (in this case, survival >3 or >12 months).

First-degree associates of survival (those most closely associated with the outcome) differed between the 2 models [4]. In the BETS-3 model (Figure 1), the senior surgeon’s estimate of survival, preoperative hemoglobin concentration, absolute lymphocyte count, presence of a completed pathologic fracture, and Eastern Cooperative Oncology Group (ECOG) performance status were found to be first-degree associates. In the BETS-12 model (Figure 2), the surgeon’s estimate of survival, preoperative hemoglobin concentration, number of bone metastases, and oncologic diagnosis group were shown to be first-degree associates. Both models were internally validated using 10-fold cross-validation methods.

The purpose of this study was to externally validate the BETS-3 and BETS-12 models using an independent, international skeletal metastasis registry containing the records of patients with operatively treated skeletal metastases. Three- and 12-month rates of survival were again used as the primary endpoints. Because Bayesian classification can effectively account for data uncertainty, it can be used in the setting of missing data, as commonly occurs in large, population-based registries such as the one chosen for this study.

## Methods

### Data collection

The Scandinavian Sarcoma Group established the Scandinavian Skeletal Metastasis Registry (SSMR) in 1999 in an effort to improve the treatment of patients with skeletal metastases. The SSMR contains the records of patients with skeletal extremity metastases who were surgically treated at one of eight major Scandinavian referral centers between 1999 and 2009. Each record contains 84 demographic and clinical variables, including most of the preoperative features required to validate the BETS models. Survival was defined as the time elapsed from the date of surgery to the date of death or last follow-up. The likelihood of survival at 3 and 12 months was the outcome. This study protocol was approved by the Scandinavian Sarcoma Group. Informed consent was not required prior to using de-identified registry data.

The BETS-3 and BETS-12 models are comprised of 9 and 10 prognostic features, respectively [4]. These include: age at the time of surgery (BETS-12 model only), sex, indication for surgery (impending or completed pathologic fracture), number of bone metastases (solitary or multiple), surgeon’s estimate of survival (postoperatively, in months), presence or absence of visceral metastases, presence or absence of lymph node metastases, preoperative hemoglobin concentration (mg/dL, on admission, prior to transfusion, if applicable), absolute lymphocyte count (K/μL), and the primary oncologic diagnosis. The oncologic diagnosis was classified into 3 groups as previously described [4]. Briefly, breast, prostate, renal cell, and thyroid carcinomas, multiple myeloma, and malignant lymphoma, which are diagnoses associated with the longest median survival time, were included in Group 3; sarcomas and other carcinomas were included in Group 2; and lung, gastric, and hepatocellular carcinomas and melanoma in Group 1.

The following definitions were used in this study. An impending pathologic fracture was one in which the degree of bone and/or cortical disruption warranted prophylactic surgical stabilization to prevent fracture. A completed pathologic fracture was one in which the lesion caused a change in bone length, alignment, rotation, or loss of height as determined by imaging. Biopsy-proven or clinically obvious metastases to organs within the chest or abdomen were considered visceral metastases. Only biopsy-proven metastases to the lymph nodes were considered indicative of lymph node involvement.

Although missing data are acceptable, the validation process requires that the specific variables present within each model also be present within the validation set. To satisfy this requirement, we converted the Karnofsky performance score, which was recorded in the SSMR, to the ECOG performance score, which is used by the BETS models, in a manner described elsewhere [11]. The units of measure for each variable in the model and validation sets must also be the same. Therefore, we converted hemoglobin concentration levels, which were reported in mmol/L or g/L in the SSMR, to mg/dL using simple mathematical formulae. No other variables in the validation set required conversion.

### Assessment of the BETS models’ performance

The characteristics of the validation set were compared to those of the test set. Distributions of categorical variables were compared using the chi-square method, and the mean values of normally distributed continuous variables were compared using the Student’s *t*-test. A two-tailed α of 0.05 was considered statistically significant. Statistical analysis was performed using SAS software (version 9.2; SAS Institute, Inc., Cary, North Carolina, USA), and validation of the Bayesian models was performed using commercially available software (FasterAnalytics, DecisionQ Corp., Washington, DC, USA).

We applied data contained in the validation set to the BETS-3 and BETS-12 models, which estimated the likelihood of postoperative survival at both 3 and 12 months, for each record. Receiver-operating characteristic (ROC) curve analysis was performed, and the area under the ROC curve (AUC) served as a metric of classifier robustness and accuracy. Validation was considered successful if the AUC was greater than 0.70 and was determined a priori. A detailed analysis of incorrect estimations was also performed.

## Results

Eight-hundred and fifteen (815) records contained adequate follow-up information to establish survival at 3 and 12 months postoperatively and thus comprised the validation set. None of these records were excluded. As expected, the demographic and clinical features of patients in the validation set differed from those of patients in the training set (Tables 1 and 2). Features that differed significantly (*P* < 0.05) were age at surgery, oncologic diagnosis grouping, presence of visceral and lymph node metastases, number of bone metastases, pathologic fracture status, ECOG performance status score, and 12-month mortality. Nonsignificant differences were observed in sex, preoperative hemoglobin concentration, absolute lymphocyte count, and 3-month mortality. Most features in the validation set had varying amounts of missing data. Notable features included the surgeon’s estimate of survival (not assessed or recorded in the SSMR database), absolute lymphocyte count (missing in 84.8%), and lymph node metastases (missing in 61.7%), all of which are first- or second-degree associates of survival in both models.

**Table 1:** Comparison of categorical features between the training and validation sets

**Feature**		**Training set (n = 189)**		**Validation set (n = 815)**			***P***
		**No. of patients**	**%**	**No. of patients**	**%**	**% Missing**	
Sex^†‡^	male	85	45.0	369	45.3	0	.91
	female	104	55.0	446	54.7		
Oncologic diagnosis grouping^†‡^	1.0	52	27.3	173	21.3	0.4	.001*
	2.0	34	18.2	74	9.2		
	3.0	103	54.5	567	69.1		
Visceral metastases^†‡^	yes	114	60.3	325	39.8	6.1	<.0001*
	no	75	39.7	441	54.1		
Lymph node metastases^†‡^	yes	36	18.8	169	20.7	61.8	<.0001*
	no	153	81.2	143	17.5		
Skeletal metastases^†‡^	solitary	55	29.0	123	15.1	3.2	<.0001*
	multiple	134	71.0	666	81.7		
Pathologic fracture status^†‡^	yes	84	44.2	614	75.3	0.7	<.0001*
	no	105	55.8	196	24.0		
ECOG performance status^†‡^	0,1,2	93	49.2	558	68.5	0	<.0001*
	3,4	96	50.8	257	31.5		
Survival > 3 months^†^	yes	129	68.3	557	68.3	0	.78
	no	60	31.7	258	31.7		
Survival > 12 months^‡^	yes	79	41.8	241	29.6	0	.002*
	no	110	58.2	574	70.4		

ECOG indicates Eastern Cooperative Oncology Group; % Missing, the proportion of unknown or missing data within the validation set.

*Distributions are significantly different between the training and validation sets by the chi-square· method.

^**†**^Denotes feature of 3-month model.

^‡^Denotes feature of 12-month model.

**Table 2:** Comparison of continuous features between the training and validation sets

**Feature**		**Training set (n = 189)**	**Validation set (n = 815)**	**% Missing data from validation set**	***P***
Age at surgery (years)^‡^	Mean	62.4	66.3	0	0.0002*
	SD	13.7	12.8		
	Median	62.7	67.0		
	IQR	54.4, 72.2	58.0, 76.0		
Hemoglobin concentration (mg/dL)^†‡^	Mean	11.5	11.5	0.6	1.0
	SD	1.9	3.5		
	Median	11.4	11.3		
	IQR	10.1, 12.9	10.3, 12.6		
Absolute lymphocyte count (K/μL)^†‡^	Mean	1.2	1.2	83.8	0.48
	SD	1.3	0.74		
	Median	1.0	1.2		
	IQR	0.6, 1.5	0.8, 1.6		
Senior surgeon’s estimate of survival (months)^†‡^	Mean	10.3	N/A	100	N/A
	SD	8.6			
	Median	6.0			
	IQR	4.0, 12.0			

SD=standard deviation; IQR=interquartile range; N/A=not applicable.

^*^Distributions are significantly different between training and validation sets by two-tailed Student’s *t*-test.

^**†**^Denotes feature of 3-month model.

^‡^Denotes feature of 12-month model.

Using a cut point of 0.5, representing a 50% probability of survival, the BETS-3 model correctly classified 3-month survival in 633 of 815 (77.7%) patients, and the BETS-12 model correctly classified 12-month survival in 555 of 815 (68.1%) patients. On ROC curve analysis, the AUCs were 0.79 and 0.76, respectively, for the BETS-3 and BETS-12 models. When compared with the original cross-validation AUCs of 0.86 and 0.83 [4], this represents a nontrivial, but acceptable, 0.07-point degradation in model performance in both the BETS-3 and BETS-12 models.

**Figure 3:**
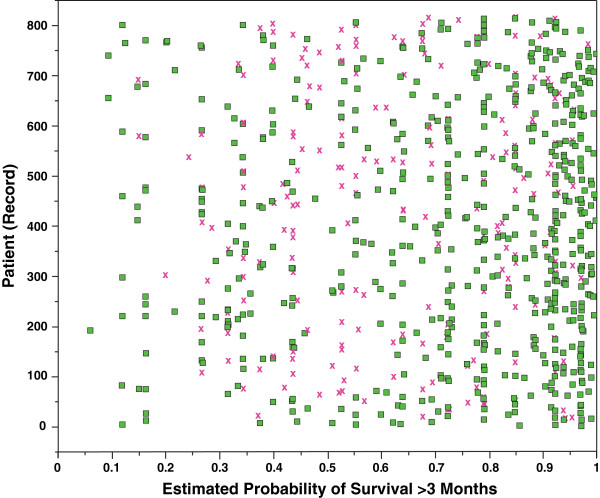
**Overlay plot of classifications made by the BETS-3 model.** This plot illustrates which records were correctly (green dot) and incorrectly (red **×**) classified as a function of the model’s predicted (estimated) probability of survival greater than 3 months. Most misclassifications were optimistic, with a median estimated probability of 0.64 (total range 0.06-1.00; interquartile range 0.44, 0.83). Three-month survival was overestimated in 142 records (incorrectly classified records from probability 0.5 to 1). In these cases, patients did not live as long as the estimated 3 months and surgery, performed at the end of life, may have been unnecessary.

**Figure 4:**
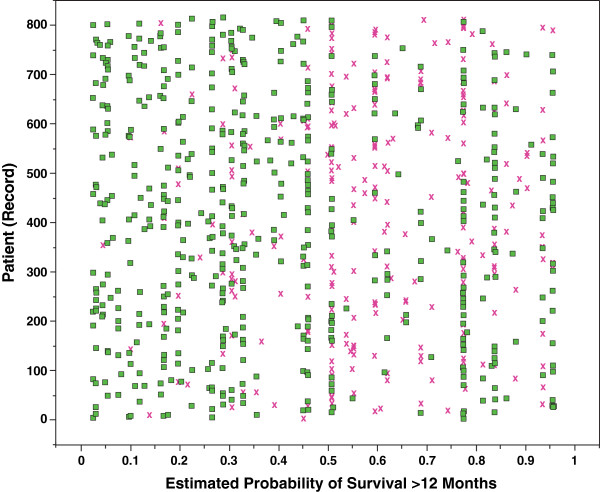
**Overlay plot of classifications made by the BETS-12 model.** This plot illustrates which records were correctly (green dot) and incorrectly (red **×**) classified as a function of the model’s predicted (estimated) probability of survival greater than 12 months. Most misclassifications were optimistic, with a median estimated probability of 0.60 (total range 0.26-0.96; interquartile range 0.51, 0.76). Twelve-month survival was underestimated in 62 records (incorrectly classified records from probability 0 to 0.5) in which patients lived longer than the estimated 12 months. They represent cases at risk for implant failure if less-invasive or less durable constructs are used.

We analyzed the records that were incorrectly classified by the BETS-3 (182, 22.4%) and BETS-12 (260, 31.9%), respectively. Of the 182 records incorrectly classified by the BETS-3 model, 125 (68.7%) were overestimates (patients did not live as long as expected) and 57 (31.3%) were underestimates (patients lived longer than expected). However, the majority (69.6%) of patients in which 3-month survival was overestimated lived greater than 1 month after surgery. Of the 260 records incorrectly classified by the BETS-12 model, 198 (76.2%) were overestimates and 62 (23.8%) were underestimates. Importantly, the majority (56.5%) of patients in whom 12-month survival was underestimated survived less than 2 years after surgery with a minority (9.6%) surviving longer than 3 years. The overlay plots (Figures 3 and 4) illustrate which records were correctly and incorrectly classified as a function of each model’s estimated probability of survival. In short, incorrect classifications made by both models tended to be optimistic, overestimating survival in most cases.

## Discussion

In this study, we successfully validated two Bayesian models previously trained to estimate the likelihood of survival at two time points that are useful for orthopaedic surgical decision making. Importantly, despite differing patient populations and varying amounts of missing data, the BETS-3 and BETS-12 models accurately classified post-operative survival at clinically useful 3- and 12-month time points.

The models performed well, despite significant differences between patients in the training and validation sets (Tables 1 and 2). Scandinavian patients in the validation set were slightly older (median age, 67.0 years [total range 23.0-96.0; interquartile range, 58.0, 76.0]) than those in the training set (median age, 62.7 years [total range 20.0-92.0; interquartile range, 54.4, 72.2]) (*P* = 0.0002). They were also nearly twice as likely to be treated for a completed pathologic fracture, as opposed to undergoing prophylactic surgery for an impending pathologic fracture (*P* < 0.0001). This may explain the significantly lower proportion of these patients surviving longer than 12 months (*P* = 0.002). Nevertheless, there were significantly higher proportions of Scandinavian patients in the more favorable diagnosis group (Group 3; *P* = 0.001) and in the more favorable ECOG performance status categories (ECOG score 0, 1, and 2; *P* < 0.001). However, there was no significant difference in 3-month survival between patients in the validation set and those in the test set (*P* = 0.78). The distributions of visceral, lymph node, and skeletal metastases also differed between the two patient populations, but this may be largely due to the proportion of missing data in the validation set.

The performance of these models is important, clinically, because inaccuracies generated by the models are not of equal significance. For example, BETS-3 was designed to identify patients that are likely to live at least 3 months who would then derive some benefit from surgery. If survival is overestimated by BETS-3, and the patient does not live at least 3 months, then the surgery may have been unnecessary. Our data show that 15.3% of records were misclassified by BETS-3 and survival overestimated, which translates to 125 potentially unnecessary surgeries performed at the end of life (Figure 3). Of these, 38 (30.4%) survived less than 1 month, 44 (35.2%) survived between 1 and 2 months and 43 (35.2%) survived between 2 and 3 months. Of course, these data do not distinguish patients who died of perioperative complications that might have been independent of the progression of disease, and in whom surgery was still the best option. Thus, surgery was still appropriate for many of the patients for whom survival was overestimated, and 15.3% represents the maximum proportion of patients who may otherwise have been spared surgery in the care of their terminal illness.

In contrast, the BETS-12 model was designed to identify patients that are expected to live 12 months or longer. This was done in an effort to help support decisions regarding the type of procedure required, as well as the durability of the implant. For example, a surgeon’s decision to perform a less invasive procedure using a less durable implant such as an intramedullary nail is supported by a low likelihood of survival at 12 months generated by the BETS-12 model. If survival is underestimated, and actual patient survival exceeds 12 months, then the chosen construct may not have sufficient durability to outlast the patient. Our results suggest that 7.6% of records may be underestimated by the BETS-12 model and misclassified in this fashion. Clinically, this represents a maximum of 62 cases at risk for implant failure that may ultimately need revision surgery (Figure 4). However, the median survival for this group of misclassified patients was 18 months [total range 12.0-73.0; interquartile range 13.8, 25.3], with 17 patients surviving longer than 24 months and only 5 surviving longer than 36 months. As such, relatively few patients, in whom the BETS-12 model underestimated survival, may have actually require revision surgery for implant failure.

Clinicians have long been interested in estimating and modeling survival in patients with metastatic cancer. For example, Bauer and Wedin [5] evaluated survival after orthopaedic stabilization in 241 patients with skeletal metastases. They found that 7 variables were independently associated with survival. Negatively associated prognostic variables included pathologic fracture, visceral or brain metastases, and a diagnosis of lung cancer, whereas positively associated variables included solitary skeletal metastases and diagnoses of lymphoma, myeloma, breast, or kidney carcinoma. Later, after retrospectively analyzing the records of 460 similar patients, the same group identified hemoglobin concentration as another negative prognosticator and discriminator of short-term survival [7]. Their work demonstrated that it was possible to make generalized estimations of survival based on disease-related and laboratory parameters; however, an accurate, individualized estimation of survival in this patient population was not possible using this method.

In an attempt to generate a prognostic tool useful for surgical decision-making, Tokuhashi et al. [12] developed a scoring system by which survival could be categorized into one of three groups: <6 months, >6 months, or >1 year. Focusing on only patients with symptomatic spine metastases, the authors collected a series of variables including, for the first time, Karnofsky performance status [13]. Other variables included were the number of extra- and intraspinal bone metastases, the number and type (resectable/nonresectable) of organ metastases, the primary oncologic diagnosis, and the degree of neurologic impairment. The group later applied their scoring system to 246 patients and found that survival greater or less than 6 months could be reliably estimated using this method [12]. Independent validation produced similar results [14]; however, this scoring system applies only to patients with symptomatic spine metastases.

Recognizing the value of a prognostic model that could be applied to all patients with skeletal metastases, Nathan et al. [15] evaluated 191 patients undergoing orthopaedic stabilization for both spine and extremity lesions. In addition to demographics, disease-specific information, and performance status [16], Nathan et al. also included a series of laboratory parameters as candidate variables. A regression-derived nomogram was developed using eight independent predictors of survival. This nomogram performed well in a small test set, but, to our knowledge, no external validation has been attempted.

We chose to use a Bayesian classifier for a variety of reasons. First, we assumed that there are, in the setting of patients with skeletal metastases, verifiable relationships between various prognostic features. The Bayesian method not only generates a joint distribution function describing the probabilistic relationships between features, but it also displays it graphically in an intuitive, transparent manner. This allows the clinician to better understand the hierarchy, and relative importance, of each feature (Figures 1 and 2) within each model. Second, Bayesian networks can account effectively for uncertainty within the data, and can thus be used in the setting of incomplete or missing input data [17]. This is a significant advantage over the traditional nomogram, when one considers that three of the first- and second-degree associates of survival—the surgeon’s estimate of survival, the absolute lymphocyte count, and the presence of lymph node metastases—were largely missing from the validation set. More importantly, the Bayesian method mimics human reasoning by updating beliefs in response to new evidence [18]. Thus, Bayesian models can be “improved” from time to time as new evidence becomes available, be it emerging patterns of disease or more effective treatment modalities. We acknowledge, however, that additional, prospective data collection is required to fulfill this goal, and we are committed to this ongoing investigation.

The BETS models discussed in this paper are clinical decision support models; their output is designed to support (not replace) good clinical judgment. The goals of surgery in patients with skeletal metastases are to relieve pain and to restore function for the maximum amount of time. Because surgery intended to relieve pain or stabilize pathologic fractures is often indicated in patients despite a very short life expectancy, a low probability of survival generated by the BETS-3 model should not be used to deny patients a palliative intervention. On the contrary, if a less invasive/less durable intervention is planned, low probabilities of survival generated by the BETS-3 and BETS-12 models would support this decision.

This study has several limitations. First, the BETS models were developed and validated using only patients who underwent orthopaedic surgery for their skeletal metastases. Thus, they are not applicable to all patients with metastatic disease or those in whom skeletal metastases were treated nonoperatively. Second, the Scandinavian patient population used for validation was well characterized and relatively homogeneous, but the generalizability of these models depends on their performance in a variety of patient populations with differing institutional biases and treatment philosophies. Finally, we believe that there is always room for model improvement, particularly when longer survival estimates are needed. Additionally, the current models are relatively optimistic, and additional covariates should be sought to help identify which patients may die earlier than expected as well as to better identify patients at risk for perioperative death. A prospective trial is currently under way to evaluate new prognostic features that may help estimate the likelihood of individual patient survival at these and other time points. Finally, the acceptance of clinical decision-support tools, such as these, depends not only on validation in additional populations, but also on how the end-user judges its availability and ease of use. It is difficult, if not impossible, to represent this classifier on paper so that other researchers may use it. To address this problem, we developed an “app” that will make this tool widely available for such a purpose.

## Conclusions

In conclusion, we successfully validated the BETS-3 and BETS-12 models using an independent, international dataset that had varying amounts of missing data per patient. These models represent the first and only validated tools for accurately estimating postoperative survival in patients with operable skeletal metastases of the extremities and can thus provide the surgeon with valuable information to support clinical decisions.

## Competing interest

None of the authors have any conflicts of interest or financial disclosures to report.

## Authors’ contributions

JF, JH, RW, HB, and PB conceived and designed the study; JF, RW, HB, BH, ML, CT, and JK collected the study data; JH, PB, HB, BH, ML, CT, and JK analyzed and interpreted the data; JF performed statistical analyses; JF and RW conducted literature searches; JF, RW, JH, and HB drafted the manuscript; and PB, BH, ML, CT, and JK critically revised the manuscript. JH, BH, ML, CT, and JK obtained study funding and JH, PB, RW, and HB supervised the study. All authors read and approved the final manuscript.

## Pre-publication history

The pre-publication history for this paper can be accessed here:

http://www.biomedcentral.com/1471-2407/12/493/prepub
